# Correction: Krymchantowski, A.V.; et al. Medication-Overuse Headache: Differences between Daily and Near-Daily Headache Patients; *Brain Sciences* 2016, *6*, 30

**DOI:** 10.3390/brainsci7030031

**Published:** 2017-03-17

**Authors:** Abouch V. Krymchantowski, Carla Jevoux, Marcelo M. Valença

**Affiliations:** 1 Headache Center of Rio, Rio de Janeiro 22031-071, Brazil; carlajevoux@uol.com.br; 2 Federal University of Pernambuco, Recife 52041-040, Brazil; mmvalenca@yahoo.com.br

We wish to make the following correction to the published paper [1]. Stewart J. Tepper did not contribute to the work presented in this manuscript and was not aware of its submission. His name is thus removed from the author list. We would like to apologize for any inconvenience caused by this change. 

## References

[1] Krymchantowski A.V., Tepper S.J., Jevoux C., Valença M.M. (2016). Medication-Overuse Headache: Differences between Daily and Near-Daily Headache Patients. Brain Sci..

